# Association between the course of hypnotics treatment for insomnia and work functioning impairment in Japanese workers

**DOI:** 10.1371/journal.pone.0243635

**Published:** 2020-12-10

**Authors:** Makoto Okawara, Tomohisa Nagata, Masako Nagata, Makoto Otani, Koji Mori, Yoshihisa Fujino

**Affiliations:** 1 Department of Environmental Epidemiology, Institute of Industrial Ecological Sciences, University of Occupational and Environmental Health, Kitakyushu, Fukuoka, Japan; 2 Department of Occupational Health Practice and Management, Institute of Industrial Ecological Sciences, University of Occupational and Environmental Health, Kitakyushu, Fukuoka, Japan; 3 Data Science Center for Occupational Health, University of Occupational and Environmental Health, Kitakyushu, Fukuoka, Japan; IRCCS Istituto Delle Scienze Neurologiche di Bologna, ITALY

## Abstract

**Study objectives:**

This cross-sectional study analyzed the effect of treatment with hypnotics for sleep disorders, particularly insomnia, on daytime work functioning by phase of treatment in Japanese workers.

**Methods:**

Subjects were respondents (n = 36,375) to a questionnaire survey conducted in 2015 to assess work functioning impairment in 15 companies in Japan. The questionnaire results were analyzed together with the respondents’ healthcare data extracted from public health insurance claims. Work functioning impairment was measured using the Work Functioning Impairment Scale (WFun). The status of treatment for insomnia was determined using data on diseases and prescribed drugs extracted from health insurance claims from the past 16 months. The odds ratio of severe work functioning impairment according to on-treatment duration and off-treatment duration was estimated using logistic regression analysis.

**Results:**

The risk of severe work functioning impairment was significantly higher in subjects with insomnia who were being treated with hypnotics for 1 month or longer compared to non-insomnia subjects. This increased risk tended to be reduced with longer on-treatment duration. For subjects who had previously received hypnotics treatment for insomnia, the risk of severe work functioning impairment was significantly increased in all subgroups stratified by time from discontinuation of the prescription. This increased risk tended to be reduced with longer off-treatment duration.

**Conclusions:**

Workers who are or were receiving hypnotics to treat insomnia may have a higher risk of daytime functioning impairment. Those with protracted insomnia require careful assessment of the risks and benefits of prescription hypnotics.

## Introduction

Sleep problems are common throughout the world, including Japan. The prevalence of sleep problems lasting 1 month or longer is approximately 20% among Japanese people [1, 2]. The economic cost of sleep disorders including insomnia in Japan is estimated to be approximately 3,500 billion Japanese yen (about 3 billion US dollars) [3].

Sleep-related problems are also an important occupational health issue. Many workers suffer from sleep problems, with an estimated prevalence of insomnia and other sleep problems in Japan of 5–45% in non-shift workers and 29–38% in shift workers [4]. Furthermore, insomnia can impair quality of daily life [5] and work performance and productivity. A meta-analysis confirmed that people with insomnia have decreased daytime cognitive function (episodic memory, problem solving, manipulation in working memory, and retention in working memory) [6]. High rates of car accidents and work accidents have been reported among people with insomnia [7]. The residual effects of some hypnotics have also been shown to be associated with car accidents [8].

Working while ill is known as presenteeism [9–11]. Sleep problems have attracted interest as one of the major conditions that can lead to presenteeism [9, 10]. For example, a study reported that insomnia patients had significantly worse health-related quality of life (HRQoL) and greater work impairment as measured by the Work Productivity and Activity Impairment-General Health (WPAI-GH) questionnaire than people without insomnia [12]. Another report showed that, compared to people who had sufficient sleep, people with insomnia experienced unintentional sleep and near misses while at work, and greater productivity loss as measured by the Work Limitations Questionnaire (WLQ) [13]. This background indicates that the health management of workers suffering from insomnia is as big a challenge as preventing the negative impact of work on sleep, such as that due to shift work and work-related stress.

Insomnia is the most common type of sleep disorder. Hypnotic drugs can be used to treat sleep disorders, especially insomnia [14, 15]. Although hypnotics can improve insomnia, they can also cause adverse effects such as daytime sleepiness and decreased mental concentration [15]. These adverse effects can arise even when using non-benzodiazepine hypnotics with a short half-life if they are inappropriately prescribed or misused. Furthermore, there are other side effects such as dependency and withdrawal syndrome, particularly for benzodiazepines, which can make it impossible to stop the prescription. For workers suffering from insomnia, important work-related issues include not only insomnia symptoms (such as nighttime sleep and daytime sleepiness) but also the maintenance of work performance (i.e., ability to concentrate on and complete tasks on time). To date, however, no studies have investigated work functioning impairment over time with regard to the course of treatment with hypnotics for insomnia.

Here, to determine the overall effect of insomnia and treatment with hypnotics on daytime work functioning in workers receiving treatment for insomnia, this study analyzed the association between the course of treatment with hypnotics (whether prescribed or not, and without regard to the duration of on- and off-treatment periods) and insomnia and work functioning impairment.

## Materials and methods

This study used data on work functioning impairment obtained from a questionnaire-based cross-sectional survey conducted in 2015 among 15 companies with 51,118 workers in Japan and healthcare data retrospectively extracted from workers’ health insurance claims. Of these 15 companies, one was in the service industry and 14 were in manufacturing. As a result of recruitment by each company, 36,375 (71%) workers from the participating companies completed a self-completion questionnaire that included questions on the individual’s demographics, job type, job title, and employment status between August 2015 and January 2016.

For workers who responded to the questionnaire, we obtained medical fee receipts (health insurance claims) from their health insurers (i.e., the companies’ health insurance associations) for the previous 16 months including the month in which the questionnaire response was provided. In Japan, all citizens are covered by the nation’s mandatory health insurance system, and all insured health care records can be obtained from health insurance claims.

### Ethics statement

The participants were told about the objectives of this study and how their data would be handled. Submission of the questionnaire survey was regarded as consent for study participation. All data were anonymized, and the researchers could not access information that could identify individual participants during or after data collection. This study, including the methods used for data collection and participant consent, was conducted after approval by the ethics committee of the University of Occupational and Environmental Health, Japan (Approval No. H26-026).

### Classification of insomnia treatment status

Subjects’ insomnia treatment status was determined based on both the diagnosis and drug prescribed, as indicated in the health insurance claims. Using data from the monthly health insurance claims for the previous 16 months, a “month with insomnia treatment” was defined as a month that met both of the following criteria: 1) registration of the disease code for “Sleep disorders” (International Classification of Diseases [ICD]-10-1 Code G47), and 2) prescription of drug(s) classified under item 112 “Hypnotics and sedatives, anxiolytics” of the Therapeutic Category of Drugs in Japan. Based on this monthly insomnia treatment status, the subjects were classified into the following three groups: an insomnia treatment continuation group (subjects who received insomnia treatment in the month in which the questionnaire response was provided and had received periodic treatment every 1, 2, or 3 months); an insomnia treatment discontinuation group (subjects who did not receive insomnia treatment in the month in which the questionnaire response was provided but had received insomnia treatment for 2 consecutive months during the 16 months prior); and a reference group (subjects without registration of a disease code for “Sleep disorders” [ICD-10-1 Code G47] during the previous 16 months). We excluded subjects (n = 2390) who had one or more missing responses in WFun (n = 612) or irregular treatment intervals (n = 1778). Because reasons for the irregular treatment intervals among subjects in the latter group were varied, including irregular visits by the patients themselves and the primary care physician allowing the patient to adjust the dose and use of medication at his or her discretion, we found it difficult to assess this group.

For subjects in the insomnia treatment continuation group, the duration of insomnia treatment (on-treatment duration) was defined as the time from the first month in which they received insomnia treatment to the month in which the questionnaire response was provided, and was classified into the following 5 categories: less than 1 month (insomnia treatment was recorded for the first time in the month in which the questionnaire response was provided; super acute phase), 1–2 months (acute phase), 3–6 months (subacute phase), 7–12 months (chronic phase), and more than 1 year (prolonged phase). We referred to previous studies on the prescription period of benzodiazepine in Japan [16].

For subjects in the insomnia treatment discontinuation group, the duration since discontinuation of insomnia treatment (off-treatment duration) was defined as the time from the last month in which they received insomnia treatment for two or more consecutive months to the month in which the questionnaire response was provided, and was classified into the following 4 categories: 1–2 months, 3–6 months, 7–12 months, and more than 1 year. If the period of discontinuation of insomnia treatment as defined above included a month in which the patient received only one month of insomnia treatment, the patient was treated as having irregular treatment intervals and was excluded from the study.

### Major outcome

Work functioning impairment was measured using WFun [17]. WFun is a self-reported outcome measure created based on the Rasch model to measure the extent of work functioning impairment due to health problems. It has been validated as consistent with Consensus-based Standards for the Selection of Health Measurement Instruments (COSMIN).

WFun assesses seven items: "I haven’t been able to behave socially," "I haven’t been able to maintain the quality of my work," "I have had trouble thinking clearly," "I have taken more rests during my work," "I have felt that my work isn’t going well," "I haven’t been able to make rational decisions," and "I haven’t been proactive about my work." Responses are provided on a five-point scale, and the degree of work functioning impairment is determined by calculating the total score. The WFun total score can range from 7 to 35, with a higher total score indicating more severe work functioning impairment. Severe work functioning impairment was defined as a WFun total score of 21 or greater, which correlated with the results of occupational health nurse interviews [18].

### Analysis

Logistic regression analysis was conducted using the on-treatment and off-treatment duration categories as independent variables, and the presence or absence of severe work functioning impairment (WFun score ≥ or < 21, respectively) as a dependent variable.

We calculated odds ratios and 95% confidence intervals for each category using subjects without registration of the disease code for “Sleep disorders” [ICD-10-1 code G47] during the previous 16 months as the reference group. Adjustment was performed for sex, age, and job type as potential confounding factors. Of the data used in the analysis, WFun score and age were treated as numerical variables, while sex, job type, and presence or absence of severe work functioning impairment were treated as categorical variables.

All statistical analyses were conducted using Stata, version 14.2 (Stata Corporation, USA). P<0.05 was considered statistically significant.

## Results

Of the 36,375 participants, we excluded 612 workers with one or more missing responses in WFun and 1,778 workers with irregular treatment intervals, leaving 33,985 respondents for analysis.

**Fig 1:**
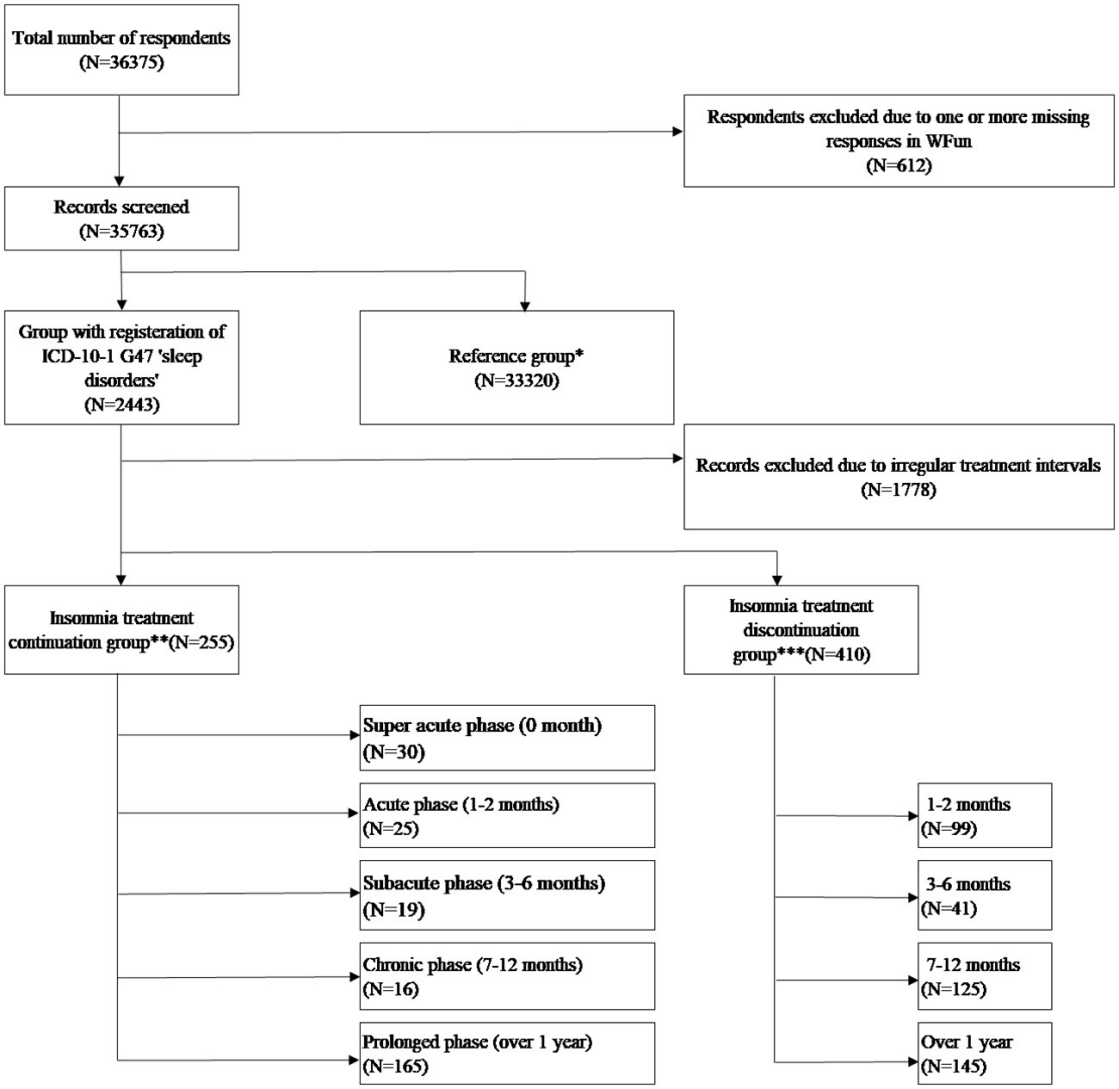
Flow chart for inclusion and exclusion of participants in the study. *ICD-10-1 G47 “Sleep disorders” was not registered in the 16-month observation period. **ICD-10-1 G47 “Sleep disorders” was registered in the 16-month observation period AND Hypnotics were prescribed monthly, once every two months or once every three months and in the month in which the questionnaire response was provided. ***ICD-10-1 G47 “Sleep disorders” was registered in the 16-month observation period AND Hypnotics were prescribed for two consecutive months in the observation period but not in the month in which the questionnaire response was provided.

The flow of subjects is shown in Fig 1.

Characteristics of the analyzed subjects are summarized in Table 1. Males accounted for 83% of all subjects, and the mean age was 40.7 years (SD = 11.2). The most common job type was production operator (24%), followed by office clerk (18%) and salesperson (16%). Managers and general employees comprised 18% and 53%, respectively, with job title data missing from 30% of the analyzed subjects.

**Table 1:** Baseline information.

	Total (n = 33985)		Reference group (n = 33320)		Insomnia treatment continuation group (n = 255)		Insomnia treatment discontinuation group (n = 410)	
Variable	n	% or mean(SD)	n	% or mean(SD)	n	% or mean(SD)	n	% or mean(SD)
Gender								
Male		83%		82%		86%		83%
Age, mean (SD)		40.7(11.2)		40.6(11.2)		44.8(9.6)		44.7(10.0)
Job type								
Office clerk	6148	18%	5976	18%	64	26%	108	26%
Salesperson	5530	16%	5418	17%	39	16%	73	18%
Researcher	2116	6%	2078	6%	18	7%	20	5%
Development worker	4244	12%	4149	13%	51	20%	44	11%
Skilled worker	1722	5%	1682	5%	11	4%	29	7%
Factory production line worker	2928	9%	2878	9%	16	6%	34	8%
Production operator	8289	24%	8184	25%	36	14%	69	17%
Other	2384	7%	2338	7%	15	6%	31	8%
Missing	624	2%	617	2%	5	2%	2	0%
Job title								
Manager	6101	18%	5957	18%	39	15%	105	26%
Non-manager	17834	52%	17459	52%	144	56%	231	56%
Missing	10050	30%	9904	30%	72	28%	74	18%
Employment status								
Permanent employee	21743	64%	21265	64%	171	67%	307	75%
Contract employee	959	3%	930	3%	8	3%	21	5%
Part-time worker	199	1%	196	1%	1	0%	2	0%
Dispatch worker	9	0%	9	0%	0	0%	0	0%
Missing	11075	33%	10920	33%	75	29%	80	20%
WFun score, mean (SD)		14.7 (6.4)		14.6 (6.3)		17.5 (7.6)		16.9 (7.2)
WFun score, median (IQR)		14 (9–19)		14 (9–19)		17 (12–22)		16 (11–22)
WFun score ≥21		20%		20%		34%		35%

The association between the duration of prescription for hypnotics and severe work functioning impairment for the insomnia treatment continuation group is shown in Table 2. Subjects whose prescription had started in the month in which the questionnaire response was provided had a lower risk of work functioning impairment, although this was not significant (OR = 0.33, 95% CI = 0.08–1.38, p = 0.127), compared to the reference group. The risk was highest for those with acute phase insomnia with an on-treatment duration of 1–2 months (OR = 5.98, 95% CI = 2.62–13.66, p<0.001). The OR for those with an on-treatment duration of 3–6 months was 3.76 (95% CI: 1.50–9.42, p = 0.005), and 3.79 (95% CI: 1.35–10.65, p = 0.012) for those with a duration of 7–12 months. For those with prolonged phase insomnia with an on-treatment duration of more than 1 year, the risk of severe work functioning impairment was significantly higher, albeit that it showed a tendency toward a gradual decrease (OR = 2.32, 95% CI = 1.66–3.24, p<0.001), compared to the reference group. The association between the duration of prescription discontinuation for hypnotics and severe work functioning impairment for the insomnia treatment discontinuation group is shown in Table 3. The risk of severe work functioning impairment was highest in those with an off-treatment duration of 1–2 months (OR = 3.31, 95% CI = 2.20–4.97, p<0.001). This increased risk tended to be reduced with longer off-treatment duration. In those with an off-treatment duration of more than 1 year, the OR for severe work functioning impairment was reduced to 2.01 (95% CI = 1.39–2.91, p<0.001), but this risk remained significantly higher than that of reference group. Among participants in the reference group, 4.2% had been prescribed at least one hypnotic medication in the previous 16 months. Results were not changed when this 4.2% were excluded from the analysis.

**Table 2:** Association between the duration of prescription for hypnotics and severe work functioning impairment (logistic regression analysis).

	n (%)	WFun score		WFun≥21	OR	95% CI		*p*
		Mean	SD	n (%)				
Reference group	33320 (99.2%)	14.6	6.3	6500 (20%)	reference			
Super acute (less than 1 month)	30 (0.09%)	12.7	5.0	2 (7%)	0.32	0.76	1.37	0.125
Acute (1–2 months)	25 (0.07%)	20.8	8.0	14 (56%)	5.96	2.61	13.61	<0.001
Subacute (3–6 months)	19 (0.06%)	19.3	10.1	9 (47%)	3.74	1.50	9.35	0.005
Chronic (7–12 months)	16 (0.05%)	20.9	7.2	8 (50%)	3.78	1.35	10.63	0.012
Prolonged (>1 year)	165 (0.50%)	17.3	7.2	54 (33%)	2.30	1.65	3.22	<0.001

Adjusted for sex, age, and job type

**Table 3:** Association between the duration of prescription discontinuation for hypnotics and severe work functioning impairment (logistic regression analysis).

	n (%)	WFun score		WFun≥21	OR	95% CI		*p*
		Mean	SD	n (%)				
Reference group	33320 (98.8%)	14.6	6.3	6500 (20%)	reference			
Discontinuation for 1–2 months	99 (0.29%)	18.6	7.4	41 (41%)	3.28	2.18	4.93	<0.001
Discontinuation for 3–6 months	41 (0.12%)	17.1	6.5	13 (32%)	2.12	1.09	4.14	0.027
Discontinuation for 7–12 months	125 (0.37%)	16.8	7.6	45 (36%)	2.66	1.84	3.86	<0.001
Discontinuation for >1 year	145 (0.43%)	15.8	6.8	43 (30%)	1.99	1.38	2.89	<0.001

Adjusted for sex, age, and job type

## Discussion

This study examined the relationship between work functioning impairment over time and the duration of prescription for hypnotics for insomnia and the duration of prescription discontinuation in Japanese workers. Our data suggest that there may be an increased risk of severe work functioning impairment in those with insomnia treated with hypnotics compared to reference subjects without sleep disorders. In addition, the increased risk of work functioning impairment tended to be reduced with longer on-treatment duration, presumably owing to improvement in insomnia by optimization of dose and drug choice in long-term treatment. However, in subjects who had a long-term on-treatment duration of more than 1 year, the risk of work functioning impairment remained high, at approximately 2.3 times that of the reference, indicating possible intractable insomnia requiring long-term prescription of hypnotics. In contrast, subjects who received temporary prescription had a lower risk of severe work functioning impairment, although this was not significant, compared to references. This lower risk in subjects who received temporary prescription may be partly explained by the use of proactive or early measures for aggressive management of sleep by health-conscious populations (e.g., prescription of a small amount of a hypnotic drug before an overseas business trip for anticipated jet lag, early consultation to seek treatment for deterioration of sleep). Alternatively, prescription of the hypnotic medication earlier in the month in which the questionnaire response was provided may have already improved the insomnia, which was then reflected in the response provided in the self-completion questionnaire.

Among the subjects in the insomnia treatment discontinuation group, the risk of severe work functioning impairment was significantly higher in all subgroups stratified by time from discontinuation of the prescription compared with the reference group. The increased risk of severe work functioning impairment tended to be reduced with longer off-treatment duration. In particular, in those with an off-treatment duration of more than 1 year, OR was reduced to approximately 2. This indicates that people for whom hypnotics can be discontinued for more than 1 year can experience an improvement in work functioning impairment. However, the risk of severe work functioning impairment in this population remained high, at approximately 2 times that of people with no sleep disorders, presumably due to the effect of primary diseases, including concurrent diseases, chronic pain, and mood disorders, that cause secondary insomnia. The risk of work functioning impairment was highest in those with an off-treatment duration of 1–2 months. Some of the subjects may have experienced rebound insomnia or a relapse of insomnia following discontinuation of oral hypnotic medication, leading to re-exacerbation of work functioning impairment.

Insomnia is strongly associated with presenteeism. According to a study by Nagata et al., the estimated cost of insomnia-related presenteeism was USD 128.26 per person per year among Japanese workers [9]. This cost is thought to include the effects of not only insomnia symptoms but also the adverse effects of hypnotics on daytime work functioning, and should be reduced by ensuring that patients receive appropriate treatment.

Hypnotics are prescribed to treat sleep disorders, and in particular presumably to reduce insomnia symptoms and daytime functioning impairment. Ideally, the patient’s condition following appropriate treatment, such as with hypnotics, and successful reduction of insomnia and daytime functioning impairment, should be equivalent to that of individuals without insomnia. However, the present study demonstrated a higher risk of work functioning impairment in both those who were receiving hypnotics and those who had discontinued hypnotics compared with people without insomnia. DiBonaventura et al. reported poorer HRQoL and WPAI scores in patients with insomnia receiving treatment with hypnotics compared to references without insomnia, and described the effect of treatment-related adverse events [12]. The beneficial effects of hypnotics in reducing insomnia on daytime activities should be balanced against their adverse effects on daytime activities.

Unnecessarily long-term continued prescription of hypnotics is not recommended in the treatment of chronic insomnia [14, 15, 19]. Consistent with this, the present study showed a higher risk of severe work functioning impairment in those receiving long-term prescription for hypnotics than references. Thus, those with protracted insomnia who are receiving treatment should be regularly re-assessed for the appropriateness of the diagnosis and treatment, as well as the risks and benefits of the prescribed hypnotics.

This study has several strengths. It is a large-scale study that used data from over 30,000 workers and their health insurance claims. The study is novel in that it shows differences in work functioning impairment among patients with insomnia based on their medication and the duration of on- and off-treatment periods. While treatment for insomnia is intended to improve daytime functioning impairment, findings from this study can aid physicians in making appropriate treatment decisions and the implementation of further studies on insomnia treatment, particularly those with a focus on improving daytime functioning.

This study has some limitations. First, the subjects were not randomized and a degree of subject bias may therefore be present. The subjects were mostly permanent employees of relatively large companies and were predominantly middle-aged or older males. This reflects the demographic characteristics of the surveyed companies, which are similar to those of the Japanese workforce as a whole. Second, in this study, WFun cannot directly assess the effectiveness of treatment of insomnia as it is affected by many other factors, including the natural course of insomnia symptoms, the effectiveness of treatment, and long-term tolerance of treatment. In addition, people who did not experience a reduction in insomnia following initiation of treatment likely progressed to a more severe condition, possibly complicated by mood disorder or other ailments, and may have suspended work or moved into retirement. Thus, we likely analyzed data only from those who had progressed through their condition relatively well. Third, this study used definitions of insomnia and hypnotics treatment based on the registered disease names from diagnoses by clinicians and hypnotics prescription data from health insurance claims. For this reason, it is possible that the subjects did not meet other diagnostic criteria for insomnia, such as the International Classification of Sleep Disorders (ICSD)-3; and sleep disorder may have been coded as a different condition that requires prescription for hypnotics for initial treatment, such as mood disorder. Therefore, the relationships identified in this study may be due to not only insomnia but also psychiatric disorders, chronic pain, or other conditions. In addition, we were unable to extract those who received hypnotics only because the classification of prescription drugs included anxiolytics. We were able to ascertain this as accurately as possible by combining the drug data with the patient’s diagnosis. Finally, we did not consider the fact that different types of hypnotics have different effect durations in this study, nor other factors that affect sleep, such as self-medication with or misuse of drugs that are not regarded as hypnotics.

## Conclusion

Patients with insomnia receiving prescription for hypnotics have an increased risk of work functioning impairment, with the risk remaining significantly high even in those with an on-treatment duration of more than 1 year; caution is required for such cases. Unnecessarily prolonged continuation of prescriptions for hypnotics is undesirable in clinical settings. Following prescription discontinuation for hypnotics, the risk of work functioning impairment is temporarily increased, but this increased risk is reduced with longer off-treatment duration.

## Supporting information

S1 File STROBE statement—Checklist of items that should be included in reports of observational studies. (DOCX)
